# Are we doing better under pressure? An audit of post surgical wounds managed with negative wound pressure therapy

**DOI:** 10.1186/1757-1146-4-S1-O48

**Published:** 2011-05-20

**Authors:** Danielle Veldhoen, Vanessa Nube, Lynda Molyneux

**Affiliations:** 1 High Risk Foot Service, Diabetes Centre, Royal Prince Alfred Hospital, Sydney, Australia

## Background

Diabetes related foot ulcers (DRFU) frequently require surgical debridement or limb saving, partial foot amputation. The resultant wounds are generally deep and associated with prolonged healing times. Negative pressure wound therapy (NPWT) is increasingly used to facilitate healing of these complex wounds. This retrospective audit provides outcome data on patients with post-surgical wounds managed with NPWT within our High Risk Foot Service (HRFS). There is a paucity of data on clinical outcomes of DRFU treated with this therapy.

## Method

Data from patients treated for post-surgical wounds within the HRFS was extracted from our electronic data base. A manual review of the patients records treated with NPWT was conducted to determine wound severity (University of Texas grading system), time to healing (from date of surgery), duration of NPWT and complications including new or recurrence of wounds and the need for further surgical management.

## Results

155 patients with post surgical wounds were managed by the HRFS between September 2006 and November 2010. Many (83%) of these were superficial ulcers (grade 1), the remainder involved tendon and or bone (Grade 2 and 3). Thirty seven patients (37 wounds) were treated with NPWT. Two were superficial (grade 1), 19 were to tendon (grade 2) and 16 were to bone (grade 3). Most (34) were clinically infected at baseline and 13 were ischaemic. Median time to healing (from the date of surgery) was 121 days. Twenty one healed and 5 cases are ongoing. NPWT was used for a median of 30 days. Two required further foot surgery before healing, 14 patients developed new ulcers (unrelated to NPWT), 3 patients who healed required below knee amputation within 2 years and 1 died during the follow-up period.

## Conclusion

NPWT is often used in our service for the management of complex post surgical wounds with close to 60% achieving complete wound closure and a low rate of major amputation. While many factors may influence this result, the increasing use of NWPT is supported in this setting. Data collection is ongoing and we plan to compare these outcomes to a historical control group and hope that this sets a benchmark for appropriate use of NPWT.

